# Integration of Augmented Reality in Temporal Bone and Skull Base Surgeries

**DOI:** 10.3390/s24217063

**Published:** 2024-11-01

**Authors:** Taku Ito, Taro Fujikawa, Takamori Takeda, Yoshimaru Mizoguchi, Kouta Okubo, Shinya Onogi, Yoshikazu Nakajima, Takeshi Tsutsumi

**Affiliations:** 1 Department of Otorhinolaryngology, Institute of Science Tokyo, 1-5-45 Yushima, Bunkyo-ku, Tokyo 113-8510, Japan; fujikawa.oto@tmd.ac.jp (T.F.); takeda.oto@tmd.ac.jp (T.T.); mizoguchi.yoshimaru@tmd.ac.jp (Y.M.); kota19951104@icloud.com (K.O.); tsutsumi.oto@tmd.ac.jp (T.T.); 2 Department of Biomedical Information, Institute of Biomaterials and Bioengineering, Institute of Science Tokyo, Tokyo 101-0062, Japan; onogi.bmi@tmd.ac.jp (S.O.); nakajima@nakajimalab.org (Y.N.)

**Keywords:** augmented reality, temporal bone surgery, 3D holographic display, surgical navigation, cognitive load, exoscope

## Abstract

Augmented reality technologies provide transformative solutions in various surgical fields. Our research focuses on the use of an advanced augmented reality system that projects 3D holographic images directly into surgical footage, potentially improving the surgeon’s orientation to the surgical field and lowering the cognitive load. We created a novel system that combines exoscopic surgical footage from the “ORBEYE” and displays both the surgical field and 3D holograms on a single screen. This setup enables surgeons to use the system without using head-mounted displays, instead viewing the integrated images on a 3D monitor. Thirteen surgeons and surgical assistants completed tasks with 2D and 3D graphical surgical guides. The NASA Task Load Index was used to assess mental, physical, and temporal demands. The use of 3D graphical surgical guides significantly improved performance metrics in cochlear implant surgeries by lowering mental, physical, temporal, and frustration levels. However, for Bonebridge implantation, the 2D graphical surgical guide performed better overall (*p* = 0.045). Participants found the augmented reality system’s video latency to be imperceptible, measuring 0.13 ± 0.01 s. This advanced augmented reality system significantly improves the efficiency and precision of cochlear implant surgeries by lowering cognitive load and improving spatial orientation.

## 1. Introduction

Critical organs in otorhinolaryngology, particularly in temporal bone and skull base surgeries, are fixed within hard tissues, preventing movement and deformation. This resulted in the early development of navigation systems for surgical assistance. Numerous studies have shown that navigation systems are useful in temporal bone and skull base surgery [1,2,3]. However, traditional navigation systems only display 2D data, leaving surgeons to mentally construct and comprehend the 3D structure. This limitation has resulted in a high demand for platforms that provide more intuitive comprehension.

The rapid advancement of augmented reality (AR) technologies has heralded a new phase in medical procedures, providing transformative solutions across a wide range of surgical fields [4,5,6]. The use of AR in head and neck surgery was shown to enhance the accuracy of intra-operative localization of anatomical structures, thereby improving surgical outcomes [7]. Despite these significant advantages, the use of AR technologies in temporal bone and skull base surgeries is limited. Conventional surgical techniques in this domain frequently rely heavily on optical instruments such as microscopes, which do not require direct line-of-sight and may not integrate seamlessly with current AR solutions that primarily use head-mounted displays (HMDs).

Our research focuses on the use of an advanced AR system that projects 3D holographic images directly into surgical footage, potentially improving surgeon orientation to the surgical field and lowering cognitive load. Previous attempts have been made to perform monitor-less surgeries using HMDs [8,9], but several issues were identified. These issues include the inability to see the surroundings due to the complete VR environment, lack of synchronization between the virtual monitor’s position and the head position due to the absence of a gyro sensor, inability to display multiple monitors, lack of functionality to display 3D holograms, and video latency. We created a system that combines exoscopic surgical footage from the “ORBEYE” and displays both the surgical field and 3D holograms on a single screen. This novel approach combines the “ORBEYE” exoscope with a cutting-edge holographic display system to improve the workflow and outcomes of otologic and temporal bone surgeries. While displaying the integrated view on the “ORBEYE” 3D monitor, we performed actual surgeries to assess the images’ resolution, usability, and practical benefits in the clinical setting. This integration aims to close a gap in current surgical practices by improving depth perception and spatial orientation while preserving the surgeon’s natural viewpoint.

## 2. Materials and Methods

**Figure 1:**
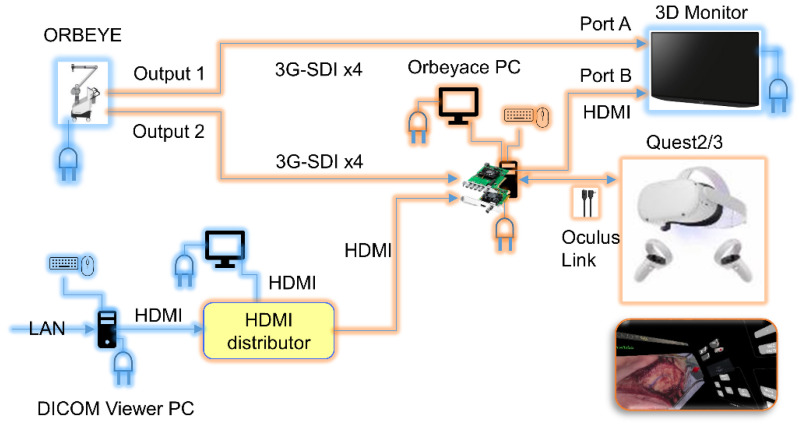
Setting up the Orbeyace holographic display system. The Orbeyace holographic display system combines 4K 3D videos from ORBEYE with a 3D monitor and the Orbeyace PC, which processes the images in Unity. The footage is then imported into the Meta Quest 2/3 headset (Meta, Menlo Park, CA, USA). Surgeons wearing the Quest 2/3 can manipulate the 3D hologram using hand gestures and integrate it with the surgical video. The combined video is sent back to the Orbeyace PC and displayed on a 3D monitor for surgical assistants and other staff to view. Additionally, images from the DICOM Viewer PC in the operating room are displayed on multiple monitors within the Quest 2/3, allowing the surgeon to view them alongside the surgical video without having to remove the headset.

*Device Configuration and Setup:* To deploy the AR system in otologic and temporal bone surgeries, one output (Figure 1, Output 1) from the ORBEYE 3D exoscope (Olympus, Tokyo, Japan) was directly connected to a 3D monitor (Figure 1, Port A), while another output (Figure 1, Output 2) was linked to the “Orbeyace” holographic display system. This setup required a number of hardware configurations, including the connections of 3D cameras and DICOM Viewer PCs via high-bandwidth digital content protection (HDCP)–compliant connections. Specifically, the Orbeyace system communicated with the ORBEYE exoscope via 3G-SDI x4 cables to capture and display high-resolution images (Figure 1, Output 2).

We chose the Video See-Through (VST) equipment over the Optical See-Through (OST) equipment due to its superior image quality and the ability to overlay high-resolution 3D holographic images directly onto the surgical field. While OST equipment is lighter, it does not provide the same level of detail and integration required for complex surgical procedures. OST displays often face challenges such as lower resolution, reduced image quality, and difficulties with color reproduction and contrast in varying light conditions, which can impact the clarity and effectiveness of holographic overlays. To ensure that the 4K image quality and resolution are maintained, we opted for VST equipment, which provides the necessary detail and integration required for our surgical applications.

*Software and Control Interface:* A hybrid interface was used to control the AR system, combining physical (mouse and keyboard) and virtual (hand gesture recognition using Quest 2/3) input methods (Figure 1). The system was run on a high-performance GPU-equipped PC that utilized the Unity platform for real-time data processing and hologram overlay. Surgeons could interact with the digital environment using hand gestures tracked by Quest 2/3 Display cameras, which allowed for the intuitive manipulation of 3D models and surgical data while maintaining sterility. This method enabled dynamic adjustments during surgery, including real-time modifications to the holographic overlays based on surgical needs.

**Figure 2:**
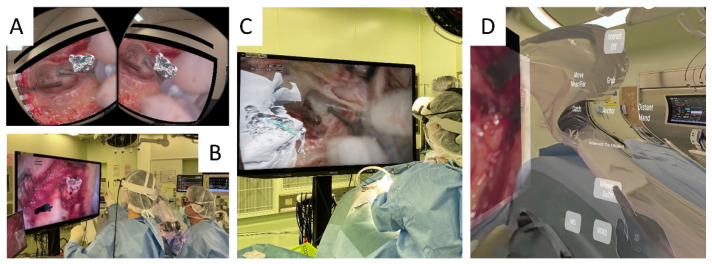
ORBEYE exoscope integrated with the Orbeyace holographic system. Using Quest 3’s color pass-through mode, surgeons can view the surgical footage in three dimensions while seeing their surroundings (**A**). When a supervising surgeon wears Quest 3, they can instruct less experienced surgeons by manipulating a 3D graphical surgical guide with hand gestures, which are displayed on a 3D monitor (**B**). Quest 3 can be worn throughout the procedure, with little noticeable latency (**C**). Figure C depicts a surgeon who appears to be looking at the 3D monitor but is actually viewing the video inside Quest 3, allowing for monitor-free surgery. Quest 3 includes various touch panels that allow the surgeon to manipulate holograms and adjust the monitor view entirely using hand gestures (**D**).

*Data Handling and Display:* The procedural data, including pre-operative imaging and intra-operative telemetry, were handled through the Orbeyace system’s software (Unity version 2021.3.22f1), which can display and manipulate complex datasets in various formats (2D, 3D, live video, and stored video). This configuration ensured continuous access to critical information, allowing for an informed surgical approach and improved cognitive support for the surgical team. The system’s ability to display multiple monitors within the HMD enabled the surgeon to view not only images captured by the 3D camera but also CT scans from the DICOM Viewer PC and pre-operative surgical simulation guides and diagrams. The footage from the Orbeyace system can be displayed on a 3D monitor via an HDMI cable (Figure 1, Port B), allowing the surgical field and 3D holograms to be viewed stereoscopically with polarized glasses, ensuring the optimal depth perception and spatial orientation required for precise surgical intervention. Using Quest 3’s color pass-through mode, surgeons can view the surgical footage in three dimensions while seeing their surroundings (Figure 2A). When a supervising surgeon wears Quest 3, they can instruct less experienced surgeons by manipulating a 3D graphical surgical guide with hand gestures, which are displayed on a 3D monitor (Figure 2B). Quest 3 can be worn throughout the procedure with little noticeable latency (Figure 2C). Quest 3 includes various touch panels that allow the surgeon to manipulate holograms and adjust the monitor view entirely using hand gestures (Figure 2D).

**Figure 3:**
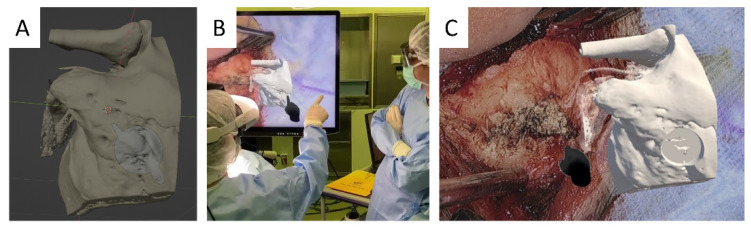
Surgical view of Bonebridge. The implantation site for the Bonebridge is pre-operatively simulated and 3D modeled using Blender, a free and open-source 3D computer graphics software toolset (**A**). Surgeons wearing Quest 2/3 can manipulate the 3D model using hand gestures (**B**) while comparing the temporal bone sutures and surface contours seen in the surgical video displayed on Quest 2/3 to the 3D model to determine the implantation site. The video in Quest 2/3 is displayed on a 3D monitor, which allows surgical assistants to view it stereoscopically using 3D glasses (**C**).

**Figure 4:**
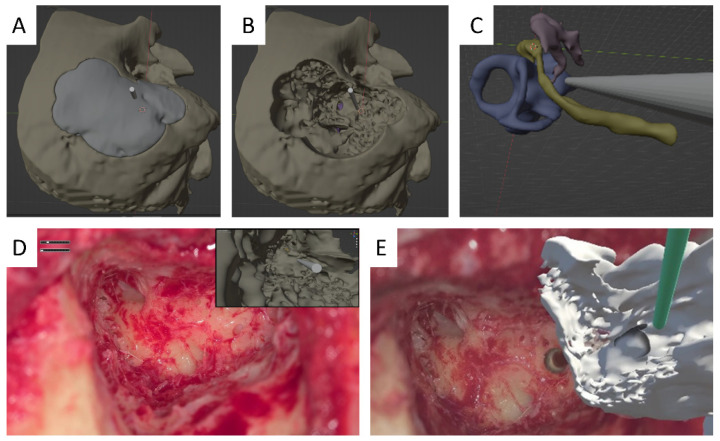
Surgical view of cochlear implant. Pre-operatively, the extent of temporal bone resection is simulated with Blender, a free and open-source 3D computer graphics software toolset (**A**,**B**). Pre-operative CT scans are used to create 3D models of the inner ear, ossicles, and facial nerve, as well as to simulate the angle at which the cochlear implant electrode can be inserted into the cochlea (**C**). (**D**,**E**) depict the surgical view following mastoidectomy. To determine the best bone resection site for inserting the cochlear implant electrode during posterior tympanotomy, the pre-operative simulation images are displayed in picture-in-picture mode on a 3D monitor for 2D viewing. For 3D display, the surgeon or supervisor wearing Quest 2/3 can manipulate the 3D model with hand gestures, which are then displayed adjacent to the surgical field to determine the resection site. The surgeon and surgical assistants examine the images on the monitor and assess the tasks using the NASA Task Load Index.

*Experimental Setup*: This study was carried out in an actual operating room during Bonebridge implantation (Figure 3) and cochlear implant surgeries (Figure 4). The primary equipment used was the ORBEYE exoscope, which was integrated with the Orbeyace holographic display system. This setup allowed for the projection of 3D holographic images directly into the surgeon’s field of view, providing real-time surgical support during surgical procedures.

*Participants*: This study included 20 surgeons and surgical assistants with varying levels of experience in Bonebridge implantation and cochlear implant surgeries, ranging from novices (residents) to experts (senior surgeons). Specifically, there were 14 novices and six experts. Some participants attended multiple surgeries, allowing them to gain a better understanding of the software through repeated exposure. Each participant completed a standard set of tasks during surgery, including orienting the surgical field for ideal bone resection angles and directions for electrode insertion in cochlear implantation, as well as identifying the implantation site for Bonebridge. These tasks utilized both 2D (Figure 3A and Figure 4A–C, and inset in Figure 4D) and 3D (Figure 3C and Figure 4E) graphical surgical guides.

*Data Collection*: The performance required to complete the tasks was quantitatively measured by the NASA Task Load Index, providing insights into each surgeon’s mental, physical, and temporal demands. Additionally, subjective feedback was gathered through structured interviews to assess the perceived benefits and challenges of 3D holographic displays as opposed to 2D graphical surgical guides.

*Procedure*: Each surgeon and surgical assistant completed the tasks twice: once with a 2D graphical surgical guide and once with the 3D holographic display. The display order was randomly assigned to avoid order effects. The tasks were designed to evaluate precision, speed, and overall comfort during the surgery. Key performance indicators such as task completion time, error rate, and precision were measured.

*Statistical Analysis*: Data were analyzed using paired t-tests to compare the performance and workload scores in the 2D and 3D conditions. The significance level was set to *p* < 0.05. Data visualization was used to show the distribution and mean differences in performance metrics.

*Ethical Considerations*: All study procedures used in studies involving human participants were in accordance with the ethical standards of the institutional research committee (M2022-072) and with the 1964 Helsinki Declaration and subsequent amendments or comparable ethical standards. All study participants provided informed consent.

## 3. Results

### 3.1. Video Latency and Surgeon Convenience/Visibility

Surgeons wearing an HMD could perform surgical procedures while viewing the surgical field at a comparable high-resolution as seen on a 4K 3D monitor, unaffected by the resolution disparity of the Quest 2/3 headset. Surgeons and assistants did not need to wear HMDs, allowing the system to be used without delay by simply switching channels from Port A to Port B. The video latency of Port B via the Orbeyace system was 0.13 ± 0.01 s, which was significantly higher than that of Port A via direct connection to a 3D monitor with 0.010 ± 0.002 s. However, all participants who experienced the system reported an imperceptible difference when performing bone drilling. The majority of surgeons did not experience dizziness during the procedures. For those who did, the symptoms were mild and temporary.

### 3.2. Quantitative Analysis

The NASA Task Load Index was used to assess subjective mental workload, and it revealed significant differences between the use of 2D (Figure 3A and Figure 4A–C and inset in Figure 4D) and 3D graphical surgical guides (Figure 3C and Figure 4E) in cochlear implant and Bonebridge implantation surgeries. Participants, including surgeons and surgical assistants, were evaluated using various metrics.

#### 3.2.1. 2D Graphic

The average scores for mental demand, physical demand, temporal pressure, performance, effort, and frustration were collected. During cochlear implant surgeries, participants reported higher mental and physical demands with average scores of 58.5 and 49.25, respectively. The temporal demand averaged 45.8, indicating moderate urgency in task completion. Performance, effort, and frustration scores were moderately high, with averages of 58.3, 54.3, and 59.0, respectively. In contrast, Bonebridge implantation had lower average scores, indicating lower demand but comparable task execution challenges.

#### 3.2.2. 3D Graphic

The use of a 3D graphical surgical guide (Figure 4E) significantly improved performance metrics in all categories during cochlear implant surgeries. Mental demand dropped to an average score of 38.3, while physical demand fell to an average score of 27.5. Temporal demand and frustration levels also improved, with average ratings of 33.5 and 31.0, respectively. Overall performance and effort improved, with average scores of 34.3 and 37.8, respectively. For Bonebridge implantation, the 3D graphical surgical guide (Figure 3C) showed no significant improvement in most metrics when compared to the 2D graphical surgical guide. In fact, the performance metric was significantly higher with a 2D graphical surgical guide (*p* = 0.045), indicating that it was superior in this regard.

#### 3.2.3. Statistical Analysis

**Figure 5:**
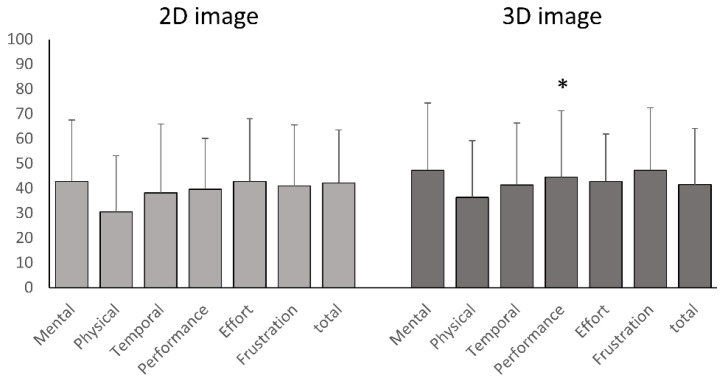
Comparison of NASA Task Load Index scores between 2D and 3D imaging for Bonebridge implantation. The assessment of subjective mental workload using the NASA Task Load Index found no significant differences between 2D display and 3D imaging technology for identifying the Bonebridge implantation site in most categories. In the performance category, 3D imaging technology received a lower rating. Asterisks (*) indicate that there is a significant difference between the scores of 2D imaging and 3D imaging. The significance was determined at *p* < 0.05.

**Figure 6:**
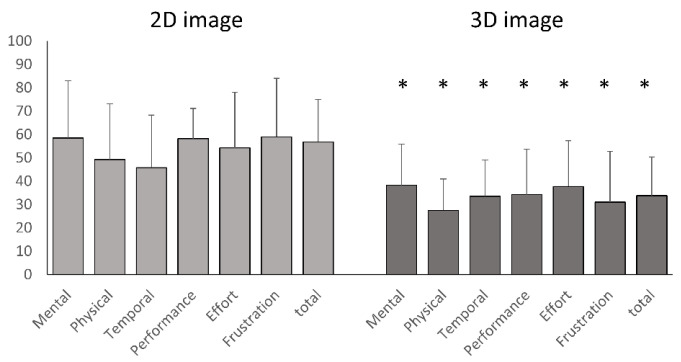
Comparison of NASA Task Load Index scores for 2D and 3D imaging during cochlear implant surgery. The evaluation of subjective mental workload using the NASA Task Load Index revealed that 3D imaging technology outperformed 2D displays in all categories for identifying the cochlear implant insertion site. Asterisks (*) indicate that there is a significant difference between the scores of 2D imaging and 3D imaging. The significance was determined at *p* < 0.05.

The reduction in scores from the 2D to 3D graphical surgical guide was statistically significant (*p* < 0.05) for all measured parameters in cochlear implant surgeries, demonstrating the superiority of the 3D graphical surgical guide (Figure 5 and Figure 6). However, for Bonebridge implantation, only the performance metric differed significantly, with the 2D graphical surgical guide performing better (*p* = 0.045), while no other metrics differed significantly between the 2D and 3D graphical surgical guides.

### 3.3. Participant Feedback

Participants’ qualitative feedback highlighted the improved spatial orientation and depth perception provided by the 3D graphical surgical guide, both of which are important factors in reducing cognitive load and improving surgical precision. This was particularly evident in cochlear implant surgeries, where the 3D graphical surgical guide significantly reduced cognitive load. In contrast, feedback for Bonebridge implantation indicated that the benefits of 3D graphical surgical guides were less pronounced, with 2D graphical surgical guides outperforming them overall in terms of cognitive load reduction.

## 4. Discussion

Most otorhinolaryngology procedures, particularly those involving the otologic and temporal bones, were traditionally performed under a microscope. During microscopic surgery, surgeons look through the eyepiece with both eyes, allowing them to work with high magnification and stereoscopic vision. To observe the surgical field from different angles, the surgeon must adjust their position alongside the microscope, which can result in awkward operating postures. The recent introduction of exoscopes has allowed surgeons to maintain comfortable postures while operating using large monitors and wearing 3D glasses, even when the camera is moved [10,11]. Exoscopes can connect to various system interfaces, making them ideal for use with AR systems. This enables the simultaneous display of multiple types of information along with the surgical field view, significantly improving surgical assistance.

The use of AR and holographic technologies in surgical procedures has significantly increased spatial awareness and precision, reducing surgeons’ mental workload. Using 3D graphical surgical guides of holographic models improves the surgeon’s ability to understand complex anatomical structures, resulting in more precise interventions [12]. Several studies have highlighted the advantages of these technologies. For example, mixed reality (MR) in surgical planning for partial nephrectomy provided a better understanding of tumor positioning, improving pre-operative planning and intra-operative decision-making [13]. Similarly, in thoracoscopic esophagectomy, holographic image-guided surgery improved safety and effectiveness by identifying anatomical variations [14]. In orthognathic surgeries such as the Le Fort I osteotomy, holograms have significantly improved surgical planning and execution accuracy [15]. Overall, incorporating AR and MR technologies into surgical practice marks a significant step forward in medical imaging and surgical support. These technologies not only improve precision and safety but also reduce the cognitive load on surgeons, resulting in better overall surgical performance and patient outcomes [12,16,17]. In our study, we integrated an AR system into the ORBEYE exoscope platform, resulting in a setup that can be completed within the monitor without the use of HMDs. Although the system had a latency of about 0.13 s, this is considered acceptable and within the tolerable range for remote surgery [18].

While the 3D holographic surgical support was extremely effective for cochlear implant surgeries, it was less useful for Bonebridge implantation. This distinction can be attributed to the unique requirements of each procedure. In cochlear implant surgeries, precise orientation is critical, and the depth and angle of bone resection have a significant impact on the success of the surgery. The 3D holographic aids in visualizing these aspects more clearly. Conversely, Bonebridge implantation requires less recognition of the surgical field’s depth and angle. The procedure consists primarily of identifying the implantation site, which can be effectively managed using 2D information. Therefore, the additional spatial information provided by the 3D holographic system has no significant impact on the surgical procedure for Bonebridge implantation.

These findings point to a promising avenue for the widespread adoption of AR technologies in medical practices. However, the integration of AR systems into surgical workflows presents several challenges and opportunities. One of the primary considerations is the creation of 3D models of organs from CT scans, which involves segmentation, modeling, and surgical simulation. This process is essential for the effective use of AR systems, as it allows for the accurate overlay of holographic images onto the surgical field. To facilitate this, the development of applications that enable surgeons to easily create these 3D models is crucial. Such applications should be user-friendly and allow for quick and accurate segmentation, modeling, and surgical simulation, ensuring that the AR system can be seamlessly integrated into the surgical workflow.

In terms of logistical considerations, it is important to develop a comprehensive platform that integrates all components of the AR system into a single, cohesive unit. This platform should include head-mounted displays and a workstation integrated with the necessary applications for creating and managing 3D models. By creating an all-in-one system, the setup and maintenance can be streamlined, reducing the need for extensive technical support and modifications to the operating room layout. This approach ensures that the AR system can be easily adopted and utilized in various surgical settings.

The concern about the unnatural positioning of hands and the patient’s surgical position when using VST equipment is valid. In our setup, the surgery is primarily performed using the ORBEYE monitor, which displays the drill, forceps, and other instruments. Whether using a monitor or an HMD, the main surgical actions remain consistent. However, for actions that are not visible on the screen, such as changing the drill tip or switching surgical instruments, there can be some discomfort when performed through the video see-through display. Despite this, otologic surgeries are mostly conducted while viewing the monitor, and the surgeon typically remains seated, minimizing the impact of this issue.

The power and transmission cables of the VST equipment are designed to be minimally intrusive. We have implemented cable management solutions to ensure that they do not interfere with the surgeons’ operations. Specifically, the cables are routed from the back of the surgeon to the PC, allowing for free movement and access to the surgical site. As above mentioned, the surgeon typically remains seated in otologic surgeries and does not move around extensively, which makes this setup manageable. While a wireless connection would be ideal to eliminate cables entirely, the large data volume and potential delays in transmission speed led us to opt for a wired solution in this instance.

An additional significant barrier is the initial cost of acquiring and implementing AR technology. Hospitals and surgical centers must weigh the benefits of AR systems against the financial investment required. Furthermore, the acceptance and adoption of AR technology by surgical teams depend on demonstrating its clear advantages in improving surgical outcomes and efficiency.

Furthermore, the current system has limitations, as it places the hologram adjacent to the surgical field for comparison. While this approach has proven sufficiently useful, we intend to refine it further to create a more intuitive system. One major limitation is the inability to align and superimpose the hologram directly on the surgical field. Overcoming this limitation will require more than just integration with existing navigation systems; it will also necessitate capturing the surgical field’s 3D structure using methods such as AR markers or 3D scanning. Addressing this limitation would allow for more accurate and intuitive visualization of the surgical area, as well as real-time, spatially accurate guidance for the surgeon.

Future research could build on this work by investigating long-term clinical outcomes and patient recovery times to fully understand the advantages of holographic surgical support in medical applications. By continuing to develop and refine these technologies, we can ensure that AR systems become an even more integral part of surgical practice, increasing procedure precision and safety and, ultimately, improving patient outcomes. To facilitate the integration of the AR system into everyday surgical practice, a comprehensive training program is necessary that includes hands-on workshops and simulation-based learning modules. This will ensure a smooth transition and proficiency in using the AR system for both surgeons and operating room staff.

## 5. Conclusions

This study demonstrates the significant potential of augmented reality (AR) technologies in improving surgical outcomes, particularly in cochlear implant surgeries. The use of 3D graphical surgical guides was shown to reduce cognitive load and enhance spatial orientation. While the benefits of 3D guides were less pronounced in Bonebridge implantation, the overall findings highlight the versatility and effectiveness of AR in various surgical contexts.

The integration of AR systems, such as the one developed in this study, represents a promising advancement in surgical technology. By providing real-time, 3D holographic images directly into the surgical field, these systems can significantly reduce the cognitive burden on surgeons and improve their spatial awareness.

Future research should focus on refining these AR systems to further enhance their usability and effectiveness. This includes addressing current limitations, such as the inability to superimpose holograms directly onto the surgical field, and exploring clinical outcomes and patient recovery times. By continuing to develop and integrate AR technologies into surgical practice, we can establish a foundation for more intuitive, efficient, and safer surgical procedures. 

## Data Availability

Data are contained within the article.
